# Feather iridescence of *Coeligena* hummingbird species varies due to differently organized barbs and barbules

**DOI:** 10.1098/rsbl.2021.0190

**Published:** 2021-08-25

**Authors:** Marco Giraldo, Juliana Sosa, Doekele Stavenga

**Affiliations:** ^1^ Biophysics Group, Institute of Physics, University of Antioquia, Colombia; ^2^ Surfaces and Thin Films, Zernike Institute for Advanced Materials, University of Groningen, The Netherlands

**Keywords:** *Coeligena*, barbules, spectrophotometry, angular reflectance, electron microscopy

## Abstract

Hummingbirds are perhaps the most exquisite bird species because of their prominent iridescence, created by stacks of melanosomes in the feather barbules. The feather colours crucially depend on the nanoscopic dimensions of the melanosome, and the displayed iridescence can distinctly vary, dependent on the spatial organization of the barbs and barbules. We have taken the genus *Coeligena* as a model group, with species having feathers that strongly vary in their spatial reflection properties. We studied the feather morphology and the optical characteristics. We found that the coloration of *Coeligena* hummingbirds depends on both the Venetian-blind-like arrangement of the barbules and the V-shaped, angular arrangement of the barbules at opposite sides of the barbs. Both the nanoscopic and microscopic organization of the hummingbird feather components determine the bird's macroscopic appearance.

## 1. Introduction

Males of many hummingbird species have highly iridescent gorget and crown patches that are involved in courtship, aggressive and territorial displays [1,2]. Bird feathers consist of a central rachis that bears ramifications (barbs) that have side branches (barbules). Stacks of melanosomes in the barbules, made up of melanin, keratin and air, create the bright colours of hummingbird feathers [3,4]. The coloration depends on the number and dimensions of melanosome layers, which vary between species and the body location of the feathers [5,6]. The gorgets of *Calypte anna* and *Coeligena helianthea* feature blue, red and violet colours that could be well explained using an effective medium multilayer model [7,8]. Similar modelling explained the feather coloration of an extensive array of hummingbird species [6].

Hummingbird iridescence offers a remarkable illustration of how behavioural and environmental conditions are key to an efficient signal transmission system, as the colour signal is only strikingly evident at specific angles of observation and illumination [9]. Interestingly, the colour appearance of the feathers during displays is not uniquely due to the morphology, but also depends on a combination of behavioural and environmental conditions [2,10]. The agonistic and courtship displays are trained, because the relative position of the signalling hummingbird with respect to the sun is crucial for the colour appearance [1,2,10]. For instance, the flashy male *Calypte anna* and *Calypte costae* show their colourful gorgets facing the sun, while *Selasphorus platycercus* tends to exhibit a more consistently reflective coloration by following a uniform spatial displacement pattern [10,11].

In our previous study on *C. anna*, we were intrigued by the finding that the barbules of the gorget feathers are arranged as a Venetian blind (i.e. a window screen in which the slats can assume different angles to partially or totally block the light), evidently to optimize the male's courtship display in front of the female [7]. This induced us to further investigate the spatial arrangement of the hummingbird feather barbs and barbules. For a comparative study, we chose six species of the genus *Coeligena*, a group of hummingbirds that occupy the Andes from Venezuela to Bolivia.

We performed spectrophotometrical and morphological measurements on the gorget feathers. We found that, depending on the species, the barbules' lamina is rotated with respect to the plane formed by the row of barbules. Furthermore, the two rows of barbules at opposite sides of the barb are not always coplanar, but form an angle, also species-dependent. We posit that the spatial organization of the barbules importantly affects hummingbird iridescence.

## 2. Material and methods

### (a) Animals and microphotography

Feathers were obtained from museums and scientific collections. Gorget and crown patches of six *Coeligena* species (*C. violifer, C. helianthea, C. wilsoni, C. prunellei, C. iris and C. torquata*) were analysed. Intact feathers were photographed with an Olympus stereoscope (SZX16 Stereo Zoom Microscope) equipped with an Olympus SC-30 digital camera. Single barbs, mounted on a rotatable stage, were photographed with a Zeiss Universal Microscope (Zeiss, Oberkochen, Germany) using 16x/0.35 and 40x/0.85 objectives and a Kappa DX-40 (Kappa Optronics GmbH, Gleichen, Germany) digital camera.

### (b) Spectrophotometry

Reflectance spectra of intact feathers were measured with a bifurcated reflection probe using a deuterium–halogen lamp and an AvaSpec-2048 spectrometer (Avantes, Apeldoorn, the Netherlands). A white reflectance standard (WS-2, Avantes) served as a reference. Reflectance spectra were also measured from single barbules with a microspectrophotometer (MSP): an epi-illumination microscope connected with a fibre optic to the spectrophotometer. The barbules were part of single barbs glued to the tip of a glass micropipette. The light source was a xenon arc and the MSP objective was an Olympus LUCPlanFL 20x/0.45.

### (c) Electron microscopy

We applied scanning electron microscopy (SEM) using a Philips XL-30. Transversally cut feather pieces were placed on a carbon stub holder and sputtered with gold. For transmission electron microscopy (TEM), intact barbs were cut from the distal portion of the feathers and prepared following standard treatments [7].

### (d) Spatial arrangement of barbs and barbules

Dependent on the species, the barbules' lamina (L in figure 2*e*) is rotated with respect to the plane formed by the row of barbules. This angle was measured on single barbs mounted at a rotatable stage attached to the MSP. The barb was rotated in steps of 5° around the position where the reflectance of the barbule was maximal. The barbule's rotation angle followed from the position where the barbule reflectance was maximal with respect to the zero position, the plane containing the row of barbules. To estimate the angle between the barbules at both sides of the barb, we observed cut feather pieces with an Olympus stereoscope (SZX16 Stereo Zoom Microscope) equipped with an SDF PLAPO 1XPF objective and an Olympus SC-30 digital colour camera. The images were analysed with the angle tool of ImageJ.

### (e) Imaging scatterometry

To investigate the spatial far-field reflection properties of the barbs, we performed imaging scatterometry on the mounted feather pieces, positioning them at the first focal point of the ellipsoidal mirror of the imaging scatterometer [12]. Scatterograms were obtained by focusing a white light beam with a narrow aperture (less than 5°) onto an area with diameter 13 µm. The spatial distribution of the far-field scattered light was recorded with an Olympus DP70 digital camera (Olympus, Tokyo, Japan).

## 3. Results

**Figure 1.**
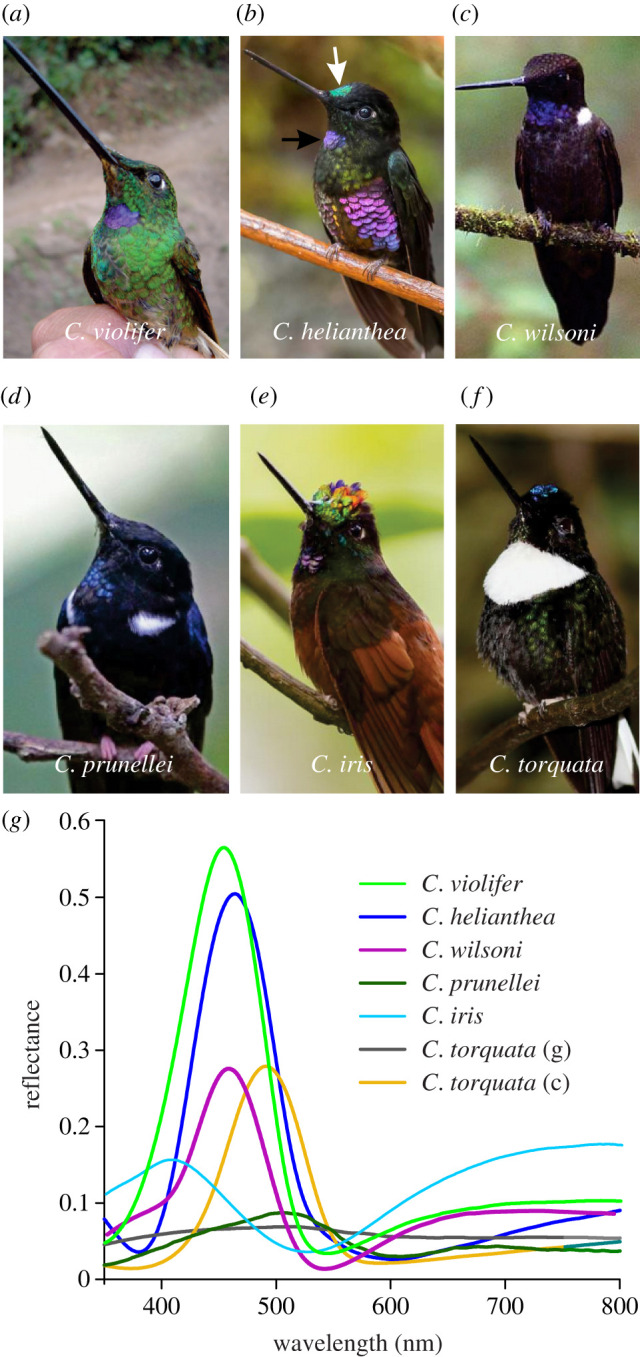
Hummingbird species of the genus *Coeligena* (males) and their coloration*.* (*a*) *C. violifer*; (*b*) *C. helianthea*; (*c*) *C. wilsoni*; (*d*) *C. prunellei*; (*e*) *C. iris*; (*f*) *C. torquata*; (*g*) reflectance spectra of the gorgets (g) of all specimens as well as of the crown of *C. torquata* (c). Gorget and crown are indicated in (*b*) with black and white arrows, respectively.

**Figure 2.**
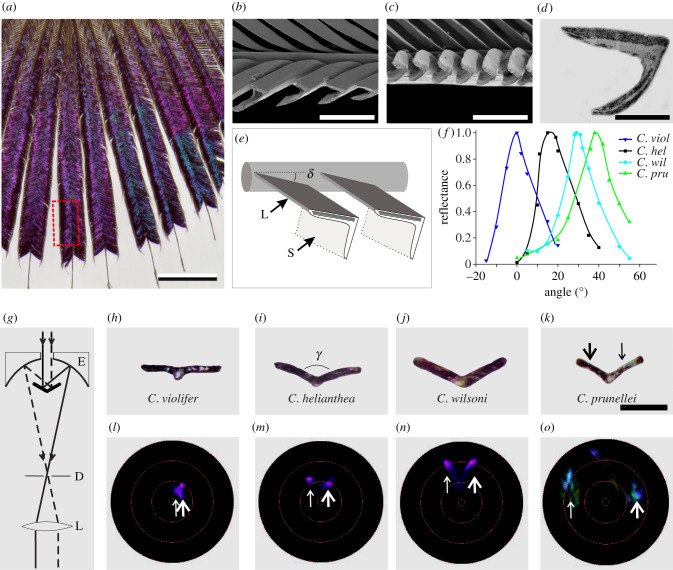
Spatial organization of the feathers [13] (electronic supplementary material, figure S1). (*a*) Microphotograph of a gorget feather of *C. helianthea*. (*b*) SEM of a longitudinal cut of the barbules of *C. violifer*, which corresponds to the dashed rectangle in (*a*). (*c*) The same for *C. prunellei*. (*d*) TEM of a sectioned barbule of *C. helianthea*. (*e*) Diagram of a barb with two barbules; L: lamina, S: side wall. The barbule lamina is rotated with respect to the barb axis at an angle *δ*. (*f*) Normalized reflectance of the barbules of *C. violifer*, *C. helianthea*, *C. wilsoni* and *C. prunellei* as a function of the angular position of the barbules. (*g*) Diagram of the scatterometer, with ellipsoidal mirror E, diaphragm D, and lens L. Two parallel light rays are incident on the two opposite barbule planes of the cut barb. (*h–k*) Cross-sections of feather barbs of a few *Coeligena* species. (*l–o*) Corresponding scatterograms. The arrows in (*k–o*) correspond to the two light rays of (*g*). The red circles in (*l–o*) indicate scattering angles of 5°, 30°, 60° and 90°. Scale bars: (*a*) 2 mm; (*b,c*) 50 µm; (*d*) 5 µm; (*h–k*) 150 µm.

The striking plumage patterns of male hummingbirds of the genus *Coeligena* display colours varying broadly across the visible spectrum (figure 1). As the gorget and frontlet, the most intensely coloured body patches, are presumably involved in courtship behaviours, we focused our study on feathers of those areas. Shiny barbules constitute the distal part of the feathers. For example, the highly iridescent gorget feathers of *C. helianthea* have barbs that feature distally purple to bluish colours (figures 1*b* and 2*a*). To quantify the optical properties of the feathers, we measured their reflectance spectra using a bifurcated reflection probe. The feathers were mounted on a stage with two rotational degrees of freedom, which was adjusted until the reflection was maximal. All the examined hummingbirds appeared to have gorget feathers with reflectance peaks between 450 and 500 nm (figure 1*g*), except for the gorget of *C. torquata*, which is whitish, yielding a very broad reflectance spectrum (figure 1*f,g*).

Measurements of reflectance spectra with a MSP indicated that the spatial arrangements of the feather barbs largely varied among the species, which we further investigated by applying microscopy and imaging scatterometry (figure 2, electronic supplementary material, figure S1). Light microscopy demonstrated that the distal part of the hummingbird feathers consists of more or less radially arranged barbs with colourful barbules on both sides (figure 2*a*). SEM revealed that the barbules resemble a folded plane (figure 2*b,c*), and TEM showed that specifically the exposed lamina (L in figure 2*e*), contains the stacks of melanosomes that act as multilayer reflectors (figure 2*d*). The barbules’ exposed laminae are rotated with respect to the plane formed by the barbules and the barb, thus together creating an arrangement as that of a Venetian blind (figure 2*b,c,e*). We measured the rotation angle (*δ*) of the barbule's exposed laminae (figure 2*e*) with respect to the barb plane using the MSP, by rotating a feather piece in steps of 5° around the position where the reflectance of the barbule was maximal. The angular position where the barb plane was horizontal, i.e. oriented normal to the MSP axis, was called 0°. The rotation angles of the barbules of *C. violifer*, *C. helianthea*, *C. wilsoni* and *C. prunellei* thus were estimated to be 0°, 16°, 29° and 39°, respectively (figure 2*f*). The spatial arrangement of the barbules on both sides of the barb also varies among the studied hummingbird species. Barb sections perpendicular to the barb axis yielded the angle between the barbule laminae on both sides, for *C. violifer*: 173°, *C. helianthea*: 135°, *C. wilsoni*: 127° and *C. prunellei*: 117° (figure 2*h–k*). The scatterograms created by illuminating barbules at opposite sides of the barb axis showed corresponding displacements of the reflected light beams (figure 2*g,l–o*).

## 4. Discussion

The reflectance spectra of *Coeligena* gorget feathers predominantly peak in the short wavelength range. The pioneering work of Greenewalt and co-workers revealed that the coloration of hummingbird feathers is structural, created by melanosome stacks [3,4]. The melanosomes’ dimensions and the relative occupation of their components (melanin, keratin and air), together with the number of melanosome layers, determine the shape and peak wavelength of the reflectance spectra [6,8]. The displayed colours can however be importantly affected by the spatial organization of the barbs and barbules, which must have severe consequences for the behavioural displays.

The reflecting Venetian blinds that are created by the barbules’ exposed laminae are set at various, species-specific angles *δ* (figure 2*e,f*), presumably for an optimal reflection of sunlight by the male's gorget feathers onto a receptive female [7]. Intriguingly, the also species-dependent angle *γ* formed by the barbules at either side of the barb stem will additionally determine the feather reflections. When *γ* approximates 180°, as in *C. violifer* (figure 2*h*), the beams reflected by the barbules at both sides of the barb will be virtually parallel, displaying a single intense flash (figure 2*l*). With a more acute angle, as in *C. prunellei*, the reflected beams will diverge, permitting two spatially separated flashes (figure 2*k*), thus reducing the iridescence. A similar diversity in the micro-organization of the barbules on the barbs exists in other bird species, e.g. in non-iridescent male tanagers [14], but it will only have severe optical consequences in the case of iridescent feathers

Highly directional iridescent colorations can be modulated by intentional movements. Hummingbirds often pursue specific lighting conditions or body orientations to enhance their conspicuousness or contrast, allowing individuals to present their coloration in either steady or flashy ways [9,10]. Consequently, the resulting complex aerial displays and communicatory repertoires can be better understood only if we know the macroscopic effects due to the mesoscopic organization of the feather's structures. Furthermore, to understand the role of colour signalling in their behavioural ecology, colour exhibition must be studied in the environmental and behavioural contexts in which they occur. For instance, male *C. costae* tend to shuttle while facing the sun, showing off their bright-purple colours [10].

Additional ecological constraints of signal propagation and the environmental aspects that condition the spatial positioning of males might favour the occurrence of complementary (e.g. acoustic and behavioural) signals, synchronized with the visual signalling. Multimodal signalling contributes to amplification or reinforcement, enhancing the transmission efficiency [11].

Both the angle *γ* between the barbules on opposite sides of the barb and the rotation angle *δ* of the barbule laminae will determine the optimal condition for specularity of the male with respect to the female. Behavioural experiments will be necessary to establish the consequences of the spatial organization of the feather barb and barbules, for courtship and also for territorial defence.

## Data accessibility

Spectrophotometry measurements for delta angle are available from the Dryad Digital Repository: https://doi.org/10.5061/dryad.qv9s4mwdn [13].
